# Differential Expression Patterns of Eph Receptors and Ephrin Ligands in Human Cancers

**DOI:** 10.1155/2018/7390104

**Published:** 2018-02-28

**Authors:** Chung-Ting Jimmy Kou, Raj P. Kandpal

**Affiliations:** Department of Basic Medical Sciences, Western University of Health Sciences, Pomona, CA 91766, USA

## Abstract

Eph receptors constitute the largest family of receptor tyrosine kinases, which are activated by ephrin ligands that either are anchored to the membrane or contain a transmembrane domain. These molecules play important roles in the development of multicellular organisms, and the physiological functions of these receptor-ligand pairs have been extensively documented in axon guidance, neuronal development, vascular patterning, and inflammation during tissue injury. The recognition that aberrant regulation and expression of these molecules lead to alterations in proliferative, migratory, and invasive potential of a variety of human cancers has made them potential targets for cancer therapeutics. We present here the involvement of Eph receptors and ephrin ligands in lung carcinoma, breast carcinoma, prostate carcinoma, colorectal carcinoma, glioblastoma, and medulloblastoma. The aberrations in their abundances are described in the context of multiple signaling pathways, and differential expression is suggested as the mechanism underlying tumorigenesis.

## 1. Introduction

The discovery of oncogenes and tumor suppressors in 1970s and subsequent advances in 1980s illuminated the mechanisms responsible for regulating the growth and proliferation of normal cells. The activation of protooncogenes and inactivation of tumor suppressors are frequently observed in cancer cells. In most cases, tumor cells display alterations in morphology, cell-cell interactions, membrane properties, cytoskeletal structure, protein secretion, and gene expression. Furthermore, transformed cells also exhibit loss of contact inhibition, self-sufficiency of growth signals, and escape from replicative senescence [1–4].

The growth and consequent metastasis of tumor cells are largely dependent on neovascularization [5], which is regulated by many different cellular signals including axon guidance molecules. Axon guiding signal molecules consist of Eph/ephrin, Semaphorins/plexins, VEGF/VEGFR, chemokines/chemokine receptors, netrins/DCC, Slit/Robo, and Notch/Delta [6]. In fact, altered abundance and regulation of these proteins have been associated with a variety of human cancers. We have focused here on Eph/ephrin molecules and their roles in tumorigenesis.

## 2. Structure of Eph Receptors and Ephrin Ligands

Eph receptors are important for development and tissue organization in multicellular organisms. These transmembrane (TM) proteins are activated by binding to ephrin ligands. Fourteen Eph receptors encoded in the human genome are divided into A and B classes. EphA receptors consist of nine members (EphA1–EphA8 and EphA10), which are activated by five different ephrin-A ligands. Five EphB receptors (EphB1–EphB4 and EphB6) bind to three ephrin-B ligands [7]. Although interactions of Eph receptors with their cognate class of ephrin ligands are well documented, interclass binding between Eph receptors and ephrin ligands has also been reported.

**Figure 1:**
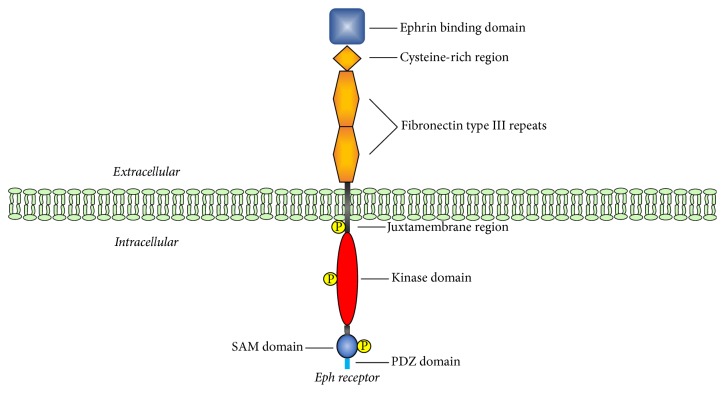
*Domains in Eph Receptors*. The cytoplasmic and extracellular portions of the receptor are separated by the membrane bilayer. The extracellular region of Eph receptors contains a ligand binding domain, a cysteine-rich domain, and two fibronectin type III repeats. The intracellular region is composed of a tyrosine domain, a sterile *α* motif (SAM), and a PDZ domain. The domains have been drawn in different shapes and colors, and individual domains are labeled with their designations. Phosphorylated residues are indicated.

The native structure of Eph receptors displays an ephrin-binding domain, a cysteine-rich region, two fibronectin type III repeats, a transmembrane segment with conserved tyrosine residues, a kinase domain, a sterile*α* motif (SAM) protein-protein interaction domain, and a C-terminal PDZ-binding motif [17–19]. The arrangement of these domains and motifs in Eph receptors is schematically represented in Figure 1. These domains and regions contribute to the 3D topology of the protein and facilitate its interaction with other proteins within the cellular signaling network. Phosphorylated amino acid residues in the activated Eph receptors mediate these interactions. However, EphA10 and EphB6 lack kinase activity due to altered sequence of the conserved regions within the kinase domain [20].

**Figure 2:**
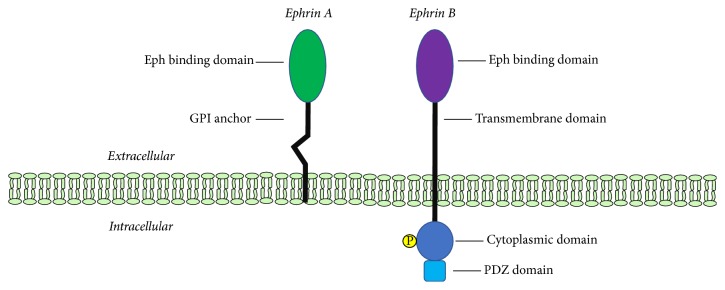
*Structure of Ephrin Ligands*. The GPI anchor and transmembrane domains of ephrin-A and ephrin-B are shown. Both classes have Eph binding domain on the extracellular side. Ephrin-B contains a cytoplasmic domain and a PDZ domain.

Eph receptors are activated by binding of ephrin ligands to the ephrin-binding domain in the receptor. The Eph binding domain of the ephrin ligand is attached to the plasma membrane by a linker segment of variable length [17]. The two classes of ligands are distinguished by the presence of GPI anchor in ephrin-A ligands and a transmembrane segment in ephrin-B ligands [21]. The structural features of the two classes of ephrins are illustrated in Figure 2.

## 3. Physiological Roles of Eph Receptors and Ephrin Ligands

The spatial organizations of Eph receptors and ephrin ligands require the presence of these molecules on the surface of two interacting cells of the same or different types. Thus, physical contact is necessary for initiating forward and/or reverse signaling in different cell types. Such contact-mediated physiological functions of these receptor-ligand pairs have been extensively documented for axon guidance, neuronal development, vascular patterning, and wound healing as described below.

### 3.1. Axon Guidance

Axons in the nervous system extend over long distances to reach their targets, and this process is facilitated by Eph receptors and ephrins. Attraction or repulsion of growth cones, which are large actin-supported extensions of a growing neurite, modulate axonal spread [112]. Interactions of ephrin-As with TrkB and p75 neurotrophin receptor lead to axon pathfinding and elongation via reverse signaling [113]. Ephrin-Bs recruit cytoskeleton regulators for axon guidance, dendrite morphogenesis, and postsynapse maturation [113]. While several other important molecules such as Zic2, neuropilin-1 (NRP1), and NrCAM are involved in guiding retinal ganglion axons, induction of EphB receptors by Zic2 transcription factor substantiate the central role of Eph/ephrin signaling in axon guidance during neurogenesis [114–119].

### 3.2. Neural Development

Neural progenitor cell proliferation, neuroblast migration, neuron survival, and neuronal plasticity also depend on Eph-ephrin interactions. The activation of EphB1, EphB2, EphB3, and EphA4 by ephrin ligands leads to migration of neuroblasts in the subventricular zone of the lateral ventricles in the adult mammalian brain [120]. Ephrin-A5 is required for the survival of newborn neurons in adult mice hippocampus, proliferation of cells in the hippocampal dentate gyrus, and the regulation of vasculature within the hippocampus [121]. Eph/ephrins also act as negative modulators in the nervous system as shown by the involvement of EphA7 and ephrin-A2 on progenitor cell proliferation in mice [122], influence of ephrin-B3 [123], and EphB3 [124] in the adult subventricular zone, and regulation of hippocampus neural progenitor growth by ephrin-A2/A3-mediated activation of EphA7 [125]. Thus, activation of Eph receptors by ephrins is critical for the maintenance, proliferation, and inhibition of neural progenitors during neurogenesis.

### 3.3. Vascular Development

EphB4 and ephrin-B2 are known for their roles in dorsal aorta and cardinal veins. The endothelial cells in the artery are marked by ephrin-B2, while EphB4 marks venous endothelial cells [126]. The interaction of ephrin-B2 in artery and Eph receptor in veins is indicative of their roles in defining boundaries between veins and arteries [127]. These observations are also confirmed by zebra fish model of vascular development [128] and mouse retinal system [129, 130].

The lymphatic vasculature, a branched network of blind-ended capillaries and collecting lymph vessels [131], requires EphB4 and ephrin-B2 to develop vascular valves to regulate unidirectional flow within the lymphatics [132]. Involvement of ephrin-B2 has been confirmed by its ability to induce VEGFR3 internalization [129] as well as lymphatic system remodeling [102, 133]. Ephrin-B2 is also necessary for blood vessel network stabilization [134, 135].

### 3.4. Tissue Injury

The healing of injured or inflamed vessels occurs by platelet plug formation and coagulation of extravasated blood. This process involves signaling pathways that facilitate the recruitment of inflammatory cells and proliferation of fibroblasts and epithelial cells. Eph/ephrin proteins partake in tissue healing as regulators of angiogenesis [19] and cell migration [136]. Eph/ephrin regulation has also been observed in renal ischemic injury [137]. Upregulation of Eph/ephrin expression in hypoxic mouse skin flap models supports the hypothesis of Eph/ephrin involvement in ischemic tissue injury repair [138]. Similarly, remodeling events following optic nerve injury in EphB3 null rodents resulted in decreased axon sprouting due to impaired interaction between macrophages and retinal ganglion cell axons [139]. Lastly, immunochemistry data showed EphB3 overexpression in invading fibroblasts and ephrin-B2 expression in astrocytes during spinal cord injury [140]. EphA4 has been implicated in the formation of astrocytic gliosis and scar formation following spinal injury in rodents and nonhuman primates [141, 142]. These observations indicate Eph/ephrin involvement in the events that follow tissue injury.

## 4. Eph/Ephrin Signaling System

Eph receptors constitute the largest family of receptor tyrosine kinases (RTK). Several features of the Eph-ephrin family distinguish it from other RTK families. RTK are activated by binding to soluble ligands, but Eph RTK bind to ephrin ligands attached to the plasma membrane of an opposing cell. Activated RTK exist as dimers, and activated Eph-ephrin signaling system exists as higher order clusters [143, 144]. The formation of multimeric structures by high affinity binding between Eph and ephrins may lead to repulsion of cells [144]. The repulsion between two cells is attributed to the cleavage of the ephrin ligand as demonstrated by the association of ADAM10 metalloprotease with EphA3 and cleavage of ephrin-A5 following its binding with EphA3 [145]. Alternatively, endocytosis of Eph-ephrin complexes by Rac-mediated actin cytoskeletal reorganization can also cause contact-mediated repulsion [146, 147]. Lastly, ephrins have the potential to elicit reverse signaling within ephrin-bearing cells [148, 149]. Although the physiological relevance of Eph-ephrin clustering is not clearly understood, it appears to determine the strength of kinase activity and the cellular response [145].

**Figure 3:**
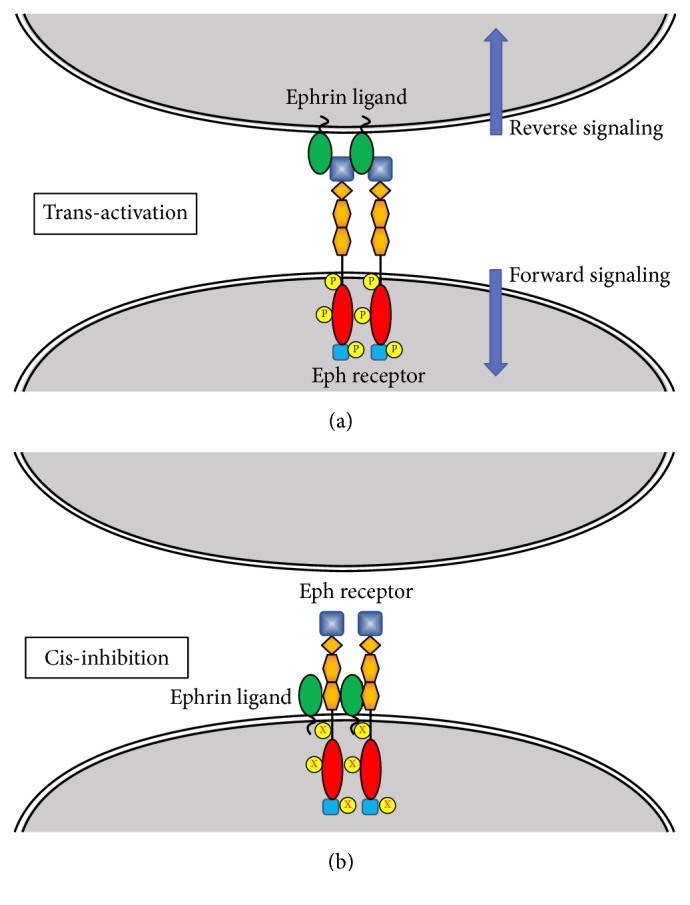
*Eph/Ephrin Forward/Reverse Signaling and Cis-Inhibition*. (a) Ephrin ligand and Eph receptors expressed on opposite cells are in trans-configuration. Both Eph receptor and ephrins activate bidirectional signaling—forward signaling with Eph receptors and reverse signaling with ephrin ligands. The activation is depicted by the presence of phosphorylated residues in the receptor. (b) Coexpression of EphA family receptor and ephrin-A family ligand on the same cell results in a cis-configuration. Such arrangement impairs Eph receptor activation and prevents trans-interaction. The inactive receptor is indicated by the lack of phosphorylated residues.

Trans-interaction between Eph receptors and ephrin ligands on opposite cells activates forward and reverse signaling. Coexpressions of EphA receptors and ephrins in their cis-interactions lead to inhibition of trans-interaction signaling [148, 150, 151].  Figure 3 summarizes the generic transactivation processes in forward signaling mediated by Eph receptors and reverse signaling mediated by ephrin ligands [113]. The figure also illustrates cis-inhibition caused by coexpression of Eph receptors and ephrin ligands in the same cell. While forward signaling involves Rho GTPases, reverse signaling is mediated by Src kinases as described below. A heterotetrameric structure is formed after binding of ephrin ligands to the glycosylated ligand binding domain of the Eph receptor, leading to the activation of the tyrosine kinase domain and subsequent phosphorylation of specific tyrosine residues [143, 152]. Activated Eph receptors recruit phosphotyrosine-binding adapters to activate Rho GTPases such as RhoA, Cdc42, and Rac for actin cytoskeleton remodeling [16]. Rho GTPases function as molecular switches that cycle between an inactive (GDP-bound) and an active (GTP-bound) state. Guanine nucleotide exchange factors (GEF) and GTPase-activating proteins (GAP) regulate the relative abundance of active and inactive Rho proteins [153]. Reverse signaling in ephrin-bearing cells begins with clustering of the ligand to promote the recruitment and activation of Src family kinases which phosphorylate specific tyrosine residues of the ligand's cytoplasmic domain [154]. The phosphorylated ligand provides a docking site for Grb4 and alters cytoskeletal dynamics by a variety of pathways triggered by several proteins such as Cbl associated protein (CAP/ponsin), Abelson interacting protein 1 (Abi-1), dynamin, paxillin, FAK, PAK1, hnRNPK, and axin [155]. Ephrin-B containing cells, on the other hand, mediate reverse signaling by recruiting intracellular adapter proteins to the phosphotyrosine residues in the cytoplasmic domain and the carboxyterminal PDZ-binding motif [156].

## 5. Eph/Ephrin Signaling in Cancer

The overexpression of several Eph receptors/ephrin ligands and downregulation of a different set of Eph/ephrin molecules in a variety of tumors suggest that these proteins have growth promoting and growth suppressing activities. Despite the challenges of resolving the complexity of Eph/ephrin signaling pathways within cancer cells, Eph receptors and ephrin ligands remain attractive targets for cancer therapy. We focus here on the mechanisms underlying the upregulation/downregulation of Eph receptors and ephrin ligands in lung, breast, brain, prostate, and colorectal cancer.

## 6. Lung Cancer

Lung cancer is the leading cause of cancer mortality in the world, with more deaths than colorectal, breast, and prostate cancer combined, and smoking is the most important risk factor in the development of pulmonary carcinomas [157]. Non-small cell lung cancer (NSCLC), a highly invasive and aggressive carcinoma, accounts for approximately 80% of all lung cancers [158]. The 5-year survival rate remains less than 15% despite the development of new surgical procedures and chemotherapeutic protocols [157, 158].

EphA2 is one of the most frequently examined Eph receptors in pulmonary carcinomas. Similarly, its ligand ephrin-A1 [159] has also been investigated in lung cancer [160, 161]. Overexpression of EphA2 in NSCLC and its correlation with smoking and metastasis [23] have been replicated in cultured bronchial airway epithelial cells (BAEpC). These studies also suggested an association of EphA2 with E-cadherin, Erk1/Erk2, p53, and JNK-MAPK pathway [24]. Overexpression of EphA2 in NSCLC patients also correlates with brain metastasis [25], and EphA2 invasive signals have been attributed in some cases to G391R mutation and consequent phosphorylation of two serine residues within mTOR [26]. The therapeutic potential of EphA2 is evident from its elevated expression in lung cancer cells that are resistant to EGFR tyrosine kinase inhibitor (TKI) and decreased viability of these resistant cells by pharmacological inhibition of EphA2 [27]. Other studies that demonstrate upregulation of ephrin-A3 in NSCLC [29] and inhibitory effects of ephrin-A3 and ephrin-B2 on transactivation of EphA2/EphA3 and EphA3/EphB4, respectively, are indicative of context-dependent aberrations of Eph/ephrin molecules in cancer cells [29, 30]. The induction of EphA3 overexpression in chemoresistant lung carcinoma cells* in vitro* has been shown to decrease chemotherapy resistance and enhance apoptosis by affecting phosphorylation of specific proteins constituting the PI3K/BMX/STAT3 signaling pathway [162]. Moderate-to-high levels of EphA4, EphA5, or EphA7 have been associated with longer survival in NSCLC patients. The combined expression of EphA1, EphA4, EphA5, and EphA7 has been used to distinguish various stages of lung cancer [22].

Among the B class of Eph/ephrins, EphB3, EphB4, ephrin-B1, and ephrin-B3 have been investigated in lung carcinoma. EphB3 overexpression is linked to clinical features of tumors and accelerated growth characteristics [31]. While* in vivo* loss of EphB3 led to activation of capase-8 and apoptosis, ligand dependent activation of EphB3 suppresses NSCLC metastasis. Mechanistically, EphB3 appears to decrease Akt activity via formation of PP2A/RACK1/Akt signaling complex [32]. Although EphB4 overexpression affects proliferation, colony formation, and motility* in vitro*, paradoxically there is a positive correlation between EphB4 expression and patient survival [33]. Cross-talk between ephrins and Eph receptors and activated status of Eph receptors have also been demonstrated by phosphoproteomic profiling of NSCLC cells. These investigations revealed that EphA2 stabilization occurs by phosphorylation of Akt in ephrin-B3 deficient NSCLC cells, and increased EphA2 correlates with worse metastatic prognosis [23, 28]. EphB6 has been shown to be prognostic indicator for NSLC [163], and deleterious mutations in this protein have also been characterized in primary tumor specimens obtained from NSLC patients [164].  Table 1 summarizes alterations in representative receptors and ligands reported by various laboratories with a tentative mechanism associated with these changes.

## 7. Breast Cancer

Eph receptors and ephrin ligands are important for mammary epithelial morphogenesis. These proteins are expressed in tumor cells as well as the tumor microenvironment, and their abundance is altered in breast carcinoma cells. We have described the following alterations in the levels of Eph receptors in breast cancer cells and briefly discussed the mechanisms underlying the expression of specific members of the Eph receptor family and their diagnostic/prognostic relevance.

EphA2 and EphB4 are the two most extensively studied receptors in breast carcinomas [23]. EphA2 is overexpressed in a majority of breast tumors, can transform normal breast cells, and is known to have both pro- and antioncogenic properties [34, 36, 165, 166]. Furthermore, expression of kinase-deficient variants of EphA2 in breast cancer cells led to decreased tumor volume and increased tumor cell apoptosis [167].* In vivo* studies have demonstrated that chronic trastuzumab treatment results in the phosphorylation of EphA2 through Src kinase, causing the activation of PI3K/Akt and MAPK pathways, which lead to trastuzumab resistance [12]. Some effects of EphA2 on tumor phenotypes are mediated by its physical and functional interaction with ErbB2/EGFR and activation of signaling pathways that involve Ras/MAPK and RhoA [35]. At cellular level, the phosphoprotein Anks1 promotes tumorigenesis by facilitating export of EphA2/ErbB2 complexes into COPII vesicles [13]. An inverse relationship between EphA2 and estrogen dependence has been observed in breast cancer cells both* in vivo* and* in vitro*, and decreased tamoxifen sensitivity was noticed in estrogen receptor (ER) positive breast cancer cells with EphA2 overexpression [168]. Exposure of ER+ breast cell lines to paclitaxel or doxorubicin also leads to increased expression of EphA2 [169]. Microarray analyses have shown a negative correlation of EphA2, EphA4, and EphA7 expression with overall survival [36]. Physical interaction of EphA7 with EphA10 [37], a kinase null receptor [46], may provide mechanistic aspects of the involvement of various Eph receptors in tumorigenesis in a context-dependent manner. Such interactions become important to explain the correlation of EphA10 expression with lymph node metastasis in breast cancer patients [38].

Among EphB receptors, EphB4 has been shown to be upregulated as well as downregulated in breast cancer cells [41, 170, 171], and knockdown of EphB4 inhibits tumor cell viability. These observations suggest EphB4 to be performing both pro- and antioncogenic roles. EphB4 expression is induced by EGFR, and inhibitors of JAK-STAT and PI3K-Akt pathways abolish EGFR induced upregulation of EphB4 receptor [41]. While antioncogenic EphB4/ephrin-B2 effects are mediated by activation of Abl-Crk pathway and downregulation of matrix metalloprotease MMP-2 [42], its tumor promoting effects manifest via ligand-independent phosphorylation [51, 52]. Additional support for EphB4 and ephrin-B2 involvement in breast cancer is provided by PP2A (protein phosphatase) knockdown effects on ERK pathway in ephrin-B2 stimulated cells [43] and morphological changes in mammary gland as well as aberrant expression of E-cadherin in mutant ephrin-B2 transgenic mice [44]. The underlying mechanism of ephrin-B2 and inappropriate E-cadherin expression may be partly explained by interactions of EphB receptors with metalloproteinase ADAM10, and subsequent E-cadherin shedding [45].

EphB6, a kinase null receptor [172] with a high affinity for ephrin-B1 and ephrin-B2 [50], has been investigated extensively for its role in breast tumorigenesis. Binding of ephrin-B1 or ephrin-B2 to EphB6 leads to its heterodimerization with EphB1, which is followed by the phosphorylation of kinase null EphB6 [46, 47]. Upon phosphorylation, EphB6 interacts with c-Cbl to promote breast tumor cell motility [48]. EphB6 expression exists in normal mammary gland and noninvasive breast tumor cell lines, but it is downregulated or absent in invasive metastatic breast cancer cell lines [173]. Levels of EphB6 are regulated by methylation of its promoter sequence in a cell-specific manner [49]. The application of methylation-dependent regulation of EphB6 expression is further evident in an investigation utilizing MSP (methylation-specific polymerase chain reaction) for potential detection of breast tumor cells in circulation [174]. Molecular and phenotypical changes in breast cancer cells appear to involve EphB6 cross-talk with cadherin 17, and altered expression of EphB6 influences WNT pathway [15]. It is noteworthy that while EphB6 has been considered a tumor suppressor in cell line models of breast tumorigenesis [15, 39, 48, 49, 173–175], its association with reduced survival in breast cancer patients has also been reported [36].

The signals transduced by the kinase-deficient EphB6 are dependent on its ability to form heterodimers with EphA2 and EphB2 [39]. Given the overexpression of EphA2 in breast cancer cells, tumor suppressor action of EphB6 may be explained by its heteromerization with EphA2 [39]. A recent study indicates the association of EphB2 expression with breast cancer survival [40]. These observations are clear indications of context-dependent biological relevance of various Eph receptors and ephrin ligands.  Table 2 summarizes altered abundance of Eph receptors and ephrin ligands with the characteristics of breast carcinoma cells.

## 8. Brain Cancer

Eph receptors have been extensively studied in glioblastoma multiforme (GBM), a subgroup of gliomas, and the pediatric brain tumor known as medulloblastoma [176–178]. While gliomas arise from astrocytes and oligodendrocytes [176, 177], medulloblastoma originate from granule neuronal precursor cells in the cerebellum or neural stem cells of the rhombic lip [178]. The migratory and invasive cell phenotype of medulloblastoma cells allow them to rapidly disseminate along leptomeningeal surfaces [179]. The involvement of Eph receptors in these two important brain neoplasms is described below.

### 8.1. Glioblastoma

EphA2 is highly expressed in GBM but not in normal brain as demonstrated by 100-fold higher levels of EphA mRNA in human GBM specimens compared to normal brain tissue [53, 180]. Particularly, EphA2 supports tumor-propagating cells with stem-like characteristics to remain in an undifferentiated state in human GBM. This has been demonstrated by the loss of self-renewal and induction of differentiation* in vitro* when EphA2 is silenced in human GBM cells via siRNA knockdown as well as ephrinA1-Fc ligand-induced EphA2 downregulation [53]. A positive correlation between EphA2 expression and pathological grade as well as proliferation has been observed in astrocytic tumors [181]. In addition, an inverse relationship exists between increased EphA2 expression and apoptosis [181]. Furthermore, a positive correlation with adverse clinical outcomes has been established with higher levels of EphA2 expression [182]. The molecular action of EphA2 in glioblastoma involves decreased Erk phosphorylation, Akt interaction, Sox downregulation, and altered invasiveness of stem cells [53–57]. These observations suggest that EphA2-mediated regulation of stemness and that of invasiveness are partly responsible for glioma phenotypes [57]. Soluble ephrin-A1, a ligand for EphA2, can lead to internalization of EphA2 and alterations in GBM cell morphology, migration, and adhesion [58, 59]. The tumorigenicity induced by EphA3, which is frequently overexpressed in the most aggressive subtype of GBM [61] but absent in normal brain tissue [62], is reduced by its ligand ephrin-A5 [63, 183]. Such effects of ephrin-A5 are attributed to an increase in the ubiquitination and subsequent degradation of the EGFR after its binding to c-Cbl [63]. Ephrin-A5 conjugated to a cytotoxin has been effective in killing GBM cells that overexpress EphA2, EphA3, and EphB2 receptors [62]. EphA3 transduces signal via MAPK pathway to maintain undifferentiated GBM cells and facilitates differentiation of neuronal progenitor cells [60, 61]. Though not well-characterized for their roles in GBM, altered expression of EphA4, EphA5, and EphA8 has been reported in GBM cells [64–68, 183]. Preliminary observations in GBM cells reveal some of these receptors as modulators of proliferation or predictors of disease status and poor prognosis [64, 66, 67, 183].

The involvement of EphB receptors in GBM cell migration and invasion and tumor angiogenesis is evident from the observations that indicate both altered abundance and phosphorylation of EphB2 and overexpression of ephrin-B3 in invasive cell lines through activation of R-Ras and Rac1 [69, 70, 75, 76]. EphB2 appears to function as a promigratory and antiproliferative molecule [71]. EphB2 is posttranscriptionally regulated by miR-204, which is downregulated in both glioma cells and neural stem cells. Given the ability of miR-204 to target SOX4, it is suggested that altered abundances of SOX4 and EphB2 together are involved in modulating stemness and migration of glioma cells [72].

Ephrin-B2 together with its receptor EphB4 promotes angiogenesis via Notch and VGFR2 [184–186] and enhances migration and invasiveness of U251 GBM cells both* in vitro* and* ex vivo* [74]. Higher expression of ephrin-B2 and EphB4 in gliomas also correlates with worse clinical prognosis [73].

The changes in Eph receptors and ephrin ligands in gliomas reported in the literature are listed in Table 3. As evident from the table, altered abundance of these molecules is brought about by different mechanisms that in turn modulate a variety of signaling molecules and pathways.

### 8.2. Medulloblastoma

Eph receptors have been implicated in vasculogenic mimicry, invasion, migration, and signaling pathways operative in medulloblastoma. EphA2 expression, in particular, is associated with phosphoinositide 3-kinase (PI3K) and vasculogenic mimicry via metalloproteinase MMP-2 [77]. Elevated expression of EphA2, EphB2, and EphB4 in medulloblastoma cell line is linked to ephrin-B1 mediated invasion [79]. The alterations in abundance and activation status of EphB2/ephrin-B1 correspond to changes in p38, Ras/Raf/Erk, PI3K, and Akt-mTOR signaling pathways [79, 187]. It is therefore not surprising that EphB2 knockdown in medulloblastoma cells combined with radiation exposure led to significant reduction of cell viability and invasion [80]. While ephrin-B1 is uniquely dysregulated in medulloblastoma, differential effects of ephrin-B1 and ephrin-B2 knockdown on phosphorylation of EphB1/B2 and Src suggest alterations in reverse signaling in medulloblastoma cells [85]. The reduction in growth and increase in radiosensitivity of medulloblastoma cells by EphB1 knockdown further substantiate the involvement of this receptor in maintaining the tumor cell phenotype [78]. A noteworthy study also demonstrates a relationship between ephrin-A5 and medulloblastoma by using a mouse model. The genetic loss of ephin-A5, a ligand for EphA4 and EphA7, led to tumor growth inhibition in a genetically engineered mouse model that harbors Smoothened gene under the control of the NeuroD2 promoter [81]. These transgenic mice have a tissue specific constitutively active form of Smoothened, which regulates ephrin-A5 expression in the dorsal midbrain and hindbrain during embryonic development of mice and chick [81, 82]. The external granule cell layer, which acts as medulloblastoma precursor, shows overexpression of ephrin-A5 [81, 83]. Molecular analysis of tumors isolated from engineered mice revealed the influence of ephrin-A5 on Akt, PI3K, and PTEN [81, 84, 188].

Table 4 summarizes variations in the levels of Eph receptors and ephrin ligands in medulloblastoma. These changes disturb relevant pathways that modulate cell proliferation, vascular reorganization, cell cycle, and tumor development.

## 9. Prostate Cancer

Prostate cancer is the third leading cause of cancer mortality in American men. A major clinical challenge in prostate cancer is distinguishing between aggressive and nonaggressive tumors [189]. Serum PSA levels have been utilized as a biomarker for over 20 years for screening and clinical management of prostate cancer [190]. However, inherent limitations of PSA screening, including a lack of specificity, have led to overdiagnosis and overtreatment of prostate cancer. Eph receptors and ephrin ligands show promise as biomarkers in many cancers and are attractive potential molecular biomarkers as well as targeted therapeutic agents for prostate cancer.

In a study consisting of cell lines representing normal prostate epithelium, primary prostate tumor, and aggressive forms of prostate tumor, several members of the Eph family were upregulated, some were downregulated, and others were either absent or unaltered. While EphA1 abundance was decreased in prostate cancer cell lines, EphA2, EphA5, EphA6, EphA7, EphA8, and EphA10 levels were elevated in some of the prostate cancer cell lines as compared to the normal prostate cell line [86]. Similar to breast cancer, EphA2 is the most extensively studied EphA receptor in prostate cancer. Early studies identified EphA2 protein overexpression in prostate cancer cell lines with greater metastatic potential, while normal and benign prostate tumor cells showed weak or no staining with EphA2 antibody [191]. A tumor grade specific increase in EphA2 protein has also been observed [87]. Stimulation of benign prostate epithelial cell line pRNS-1-1 with a soluble form of ephrin-A1 leads to decreased proliferation [192], and activation of EphA2 in PC3 cells decreases cell migration [193]. Furthermore, stimulation of EphA2 by ephrin-A1 in PTEN null PC3 cell line demonstrated inhibition of the Akt-mTORC1 pathway [34, 187, 194]. Transfection of PC3 cells with kinase-deficient mutant forms of EphA2 showed reduced metastasis when compared to PC3 cells with overexpression of native EphA2 [195]. While EphA2 dependence on ephrin ligand manifests varied phenotypic effects [192–194], overexpression of EphA2 is related to induction of metastasis [196]. It appears from these observations that EphA2 effects manifest in a context-dependent manner.

Upregulation of EphA3 in androgen independent prostate cancer cells compared to androgen dependent prostate cancer cells has been observed by microarray analysis [89], and a tentative relationship between mutant AMP-activated protein kinase (AMPK) and upregulation of EphA3 mRNA has been proposed [90]. An increase in EphA4 mRNA and protein levels has been reported when prostatic intraepithelial neoplasia progresses to prostate carcinoma, and knockdown of EphA4 has shown altered viability and colony forming ability of cancer cells [91]. In a separate study, EphA4 stimulation by ephrin-A5 resulted in inhibition of PC3 cell migration by impairment of cell-cell contact [88]. The linkage of EphA4 with prostate cancer associated receptor ERBB3/HER3 [92] is apparent from the observed decrease of EphA4 transcript following the knockdown of ERBB3 in DU145 cells [92].

The EphA receptors that are decreased or lost in prostate cancer include EphA5 in patients with a Gleason score of 8 [197], EphA6 in LNCaP-19 cell line [198], and EphA7 in prostate tumor specimens [199]. Transcriptional silencing of EphA7 in a subset of prostate cancer cells is regulated by methylation of the EphA7 promoter [199]. The presence of EphA7 in primary tumors and its loss in lymph and bone metastases suggests that promoter methylation is perhaps not an early event in prostate cancer [200]. A recent genome sequence analysis has identified a single nucleotide polymorphism (rs731174) in an intron of the EphA10 gene that may interact with other SNPs to modify prostate cancer risk [201].

The prostate cell line panel has indicated a decrease in EphB2 with elevations in both EphB3 and EphB6 in some prostate carcinoma cells compared to normal prostate epithelial cells [86]. Specimens from metastatic prostate carcinoma showed missense and nonsense mutations in the kinase domain of EphB2, and transfection of normal EphB2 in DU145 cell line led to the suppression of growth and colony formation [93]. A higher frequency of a germline nonsense mutation termed K1019X (3055A>T) has been observed in African American men as compared to Caucasian men [202, 203]. Microarray and RT-PCR analysis of prostate cancer tissue have also identified differential expression of EphB3 [204]. Several studies indicate upregulation of EphB4 in the development and progression of prostate cancer [52, 205, 206].

The literature on ephrin alterations in prostate cancer is scarce. The cell line panel indicates increased abundance of ephrin-A1 and eprin-A2 in LNCaP and DU145 cells as compared to normal cells. Ephrin-B3 was detected at higher levels in all prostate carcinoma cell lines [86]. Microdissections of prostate carcinoma samples showed lower levels of ephrin-A1 mRNA in samples with Gleason score > 7 and higher mRNA levels of ephrin-A1 from samples with Gleason score < 7 [94]. In light of the decreased migration of prostate cancer cells upon stimulation of EphA2 with ephrin-A1, downregulation of ephrin-A1 in aggressive prostate cancers is not surprising. Increased levels of ephrin-A5 in LNCaP cell culture media after androgen exposure suggests androgen-induced release of ephrin-A5 from prostate cancer cells [95]. Additionally, an independent retrospective study on metastatic castration-resistant prostate cancer has reported a correlation of lower serum levels of ephrin-A5 with shorter survival time [96].

The significant alterations of Eph/ephrin profiles observed in prostate tumors and prostate cancer cell lines are listed in Table 5. It warrants mention that the molecular changes in prostate cancer cells are also responsive to their dependence on androgen.

## 10. Colorectal Cancer

Colorectal cancer (CRC) is the third most commonly diagnosed cancer in both men and women and is the fourth leading cause of cancer-related death worldwide [207]. About 5% of CRC are monogenic, which include Lynch syndrome, familial adenomatous polyposis (FAP), MYH-associated polyposis, and rare hamartomatous polyposis syndromes [208]. Several Eph receptors and ephrin ligands exist as a gradient along the colon crypt axis of normal tissue [209]. While EphB1, EphB2, EphB3, EphB4, EphB6, EphA1, EphA4, and EphA7 are abundant in the basal crypt, the top of the crypt displays EphA2, EphA5, ephrin-A1, and ephrin-B2 [209].

The relationship of elevated expression of EphB2 and EphB3 with abnormal migration of epithelial cells in the crypt villus junction in colon tumors of mice is suggestive of Eph receptor involvement in colorectal cancer [210]. Immunohistochemical analyses have revealed decreased abundance of EphA6, EphA7, and EphB1 in colorectal tumors [211]. The expression of EphB2, an important molecule responsible for correct positioning of epithelial cells in the crypt [209], is reduced in CRC [212, 213], and its higher expression is associated with prolonged survival of CRC patients [210, 213–215]. The altered expressions of EphB2 and EphB4 in colorectal cancer have been explained by changes in adenomatous polyposis coli (APC) suppressor gene activity, CBP complex, and Wnt pathway [104, 105, 216]. Transcriptional silencing or downregulation of specific Eph receptors in CRC is associated with promoter methylation [97, 103, 211]. The activation of EphB3 in HT-29 human colon cancer cells inhibits epithelial-to-mesenchymal transition via cell adhesion molecules [106, 107]. The elevated levels of EphB4 in CRC [104, 108] are being utilized for image guided colorectal surgery [109]. Although reduced abundance of EphB6 in CRC correlates with poor cell differentiation, advance disease, and poor prognosis [110], the mechanisms of EphB6 involvement in CRC are not well understood [111].

While the expression of EphA1 and EphA2 increases in early stages of CRC, the abundance of these receptors decreases in advanced stages of the cancer [97–99, 101]. The linkage of decreased EphA1 levels with higher invasiveness is supported by alterations in adhesion and motility of HRT18 CRC cells that had been rendered EphA1 null by gene knockout [100]. The alterations of Eph/ephrin profiles of colorectal tumors and cell lines described in this section are summarized in Table 6.

## 11. Conclusion

**Figure 4:**
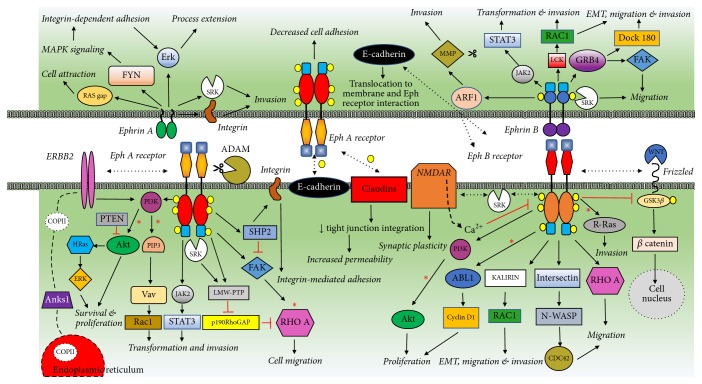
*Summary of Potential Eph/Ephrin Tumor Promoting Pathways*. A composite scheme of major tumorigenesis promoting Eph/ephrin signaling pathways is shown together. Ligand-independent forward signaling tumor promoting pathways shown for EphA and EphB receptors. Forward signaling pathways marked with an asterisk are known to be inhibited in ligand-dependent manner and participate in tumor suppression. Reverse signaling pathways are also shown for ephrin A and ephrin B. Yellow circles indicate phosphorylation of specific tyrosine/serine/threonine residues that are required for pathway activation. The broken bidirectional arrow represents cross-talk between Eph/ephrin and other types of receptors or pathways. Scissors symbol represents expression and/or function of proteases such as ADAM or MMP that are involved in the regulation of EphA and ephrin-B pathways, respectively. EMT indicates epithelial-to-mesenchymal transition. The figure is adapted from representative publications of Pasquale [8], Lisle et al. [9], Boyd et al. [10], and Xi et al. [11]. In addition, some of the pathways are substantiated from observations presented in several reports in the literature related to trastuzumab [12], COPII vesicles [13], NMDA receptor [14], E-cadherin [14], WNT pathway [15], and claudins [14, 16] for their relevance to tumor promoting pathways.

Based on the literature presented in this review, a composite network emerges that connects numerous pathways (Figure 4). This scheme was composed by adapting individual pathways described by other investigators [8–11]. The supporting data for other pathways and cross-talk among individual players is described in several publications, a few of which are cited here [12–16]. Thus, the involvement of Eph receptors and ephrin ligands in such a complex network illustrates aberrant regulation of these important molecules in tumorigenesis. It also suggests the mechanisms underlying cancer cell phenotypes associated with aberrant expression of Eph receptors.

The description presented in this review clearly demonstrates that elevated expression and/or loss of expression of specific Eph receptors are associated with either tumor growth or tumor suppression in a context-dependent manner. We suggest these consequences to arise by interaction of phosphorylated receptors with distinct intracellular proteins involved in pathways that either promote or inhibit cell proliferation and actin organization. The investigations on protein-protein interactions indicate that kinase-deficient Eph receptors, EphB6 and EphB10, can heteromerize with kinase sufficient receptors. Specifically, EphB6 heteromerizes with EphA2 and EphB2, and EphA10 interacts with EphA7 [37, 39]. Such interactions in different contexts are likely to mediate different cancer phenotypes.

Mechanistically, cis-interaction of Eph receptors with ephrin ligands can inhibit transactivation-mediated tumor suppression activity [30, 150, 217]. Alternative mechanisms of tumorigenesis include activating oncogenic mutations or inactivating mutations in tumor suppressor functions of Eph receptors, regulation of epithelial-mesenchymal transition (EMT), control of motility and invasiveness, and alterations in Akt and MAP kinase pathways [26, 34, 57, 218–221]. All these modalities of transformation include Eph receptor functionality or lack thereof. EMT, a critical aspect of cell migration, accompanies ligand-independent signaling, while ligand-dependent forward signaling restores cell-to-cell communication [219, 220]. A significant involvement of Eph receptors in tumorigenesis is based on their roles in regulating stemness of a subpopulation of cancer cells that are largely responsible for resistance to therapy [53, 61, 222]. In light of these observations, investigations on Eph receptor-mediated self-renewal of cancer stem cells are gaining momentum. The ability of Eph receptors to stimulate T cells has highlighted their importance in developing cancer immunotherapy [223–228].

The therapeutic applications of Eph receptors include monoclonal antibody targeting, soluble Eph fusion protein targeting, small molecule Eph kinase inhibitors, dendritic-cell based vaccines, and siRNAs [10, 229–233]. However, these therapeutic modalities suffer from deficiencies such as varying effectiveness of antibodies, deleterious side effects, redundancy of functions, receptor-independent activation of signaling pathways, variable effects of Eph receptors in T-cell lineage development, and epigenetic regulation of Eph expression [61, 97, 229–241].

A potential therapy for cancer cells can be tailored around a synthetic Notch (synNotch), which would allow engineered cells to respond to multiple stimuli with distinct transcriptional programs [242]. Such engineered synNotch construct consisting of Eph/ephrin would be expected to emulate contact induced cis-inhibition in tumor cells. In light of the involvement of cancer stem cells (CSC) in metastasis [222] and the importance of Eph receptors in CSC maintenance [243], Eph/ephrins are important targets for therapeutic exploration. Illustrative examples of Eph receptors in stemness include the effects of EphA3 knockdown on GBM cell sphere formation [61] and the regulation of oncogenic Ras by EphA2 in transformed cells as well as expulsion of these cells from stem cell monolayer [244, 245].

The literature reviewed here clearly presents a common theme of tumorigenesis for various human cancers that involves a set of Eph receptors and ephrin ligands. Although some of these molecules appear to be facilitating similar processes in all cancers, the differences in the outcomes in certain situations may be attributed to the context and redundant expression of specific sets of Eph receptors and ephrin ligands. Further study in how mechanistically cancer cells initiate Eph cis-signaling, the role of Eph RTK in maintaining cancer-like stem cells within the microenvironment, and the extent of Eph functional redundancy may be beneficial in overcoming the challenges of developing targeted Eph therapy to combat the tumorigenic pathway.

## Figures and Tables

**Table 1:** Altered expression of Eph receptors and ephrin ligands in lung cancer.

Eph receptor/ephrin ligand	Preferred molecular interaction	↑/↓ relative to normal tissue	Mechanism	References
EphA1		↑ (NSCLC)	(i) Higher levels of EphA1, Eph4, EphA5, and EphA7 only present in nonadvance stages of lung cancer (E-COG performance score < 2)	[22]

EphA2	Ephrin-A1	↑ (NSCLC)	(i) Tobacco smoke → upregulates EphA2 and downregulation of E-cadherin (1) Increased protein permeability via Erk1/Erk2, p54, and JNK-MAPK (ii) Highest EphA2 in patients who developed brain metastasis (iii) Constitutive activation via phosphorylation of pSer^2448^ and pSer^2481^ in MTOR (iv) Loss of EphA2→ loss of EGFR mutations in vitro in EGFR^M790M^ TKI resistant lung cancer cells (v) Phrenological inhibition of EphA2 via AWL-II-41-247 resulted in decreased proliferation of TKI resistant lung cancer cells (vi) Ephrin-B3 silencing→ stabilization of EphA2 via phosphorylation of Akt target Ser-897	[23–28]

EphA3	Ephrin-B2	↓ (SCLC)	(i) Upregulation of EphA3 → reduced PI3k/BMX/STAT3 signaling in SCLC cells → inducing G0/G1 arrest and increased apoptosis (ii) Ephrin-A3 coexpression inhibits EphA3 trans activation (1) Ephrin-A3 is 26x upregulated in NSCLC & absent in normal lung tissue	[29, 30]

EphA4		↑ (NSCLC)	(i) EphA4 expression correlate with low stage and presence of inflammation (ii) Higher levels of EphA1, Eph4, EphA5, and EphA7 only present in nonadvance stages of lung cancer (E-COG performance score < 2)	[22]

EphA5		↑ (NSCLC)	Higher levels of EphA1, Eph4, EphA5, and EphA7 only present in nonadvance stages of lung cancer (E-COG performance score < 2)	[22]

EphA7		↑ (NSCLC)	(i) EphA7 expression correlate with older age, fibrosis, and smaller tumor size (ii) Higher levels of EphA1, Eph4, EphA5, and EphA7 only present in nonadvance stages of lung cancer (E-COG performance score < 2)	[22]

EphB3	Ephrin-B1 Ephrin-B2	↑ (NSCLC)	(i) Overexpression of EphB3→ accelerated cell growth and migration (ii) EphB3/Ephrin-B1→ decreased Akt activity via ternery formation of PP2A/RACK1/Akt (iii) EphB3 loss in vivo→ apoptosis due to increased activation of capase-8	[31, 32]

EphB4		↑ & ↓ (paradoxical)	(i) 3x overexpression in lung cancer (ii) Positive correlation of EphB4 expression and lung cancer survival	[33]

Ephrin-A3		↑	(i) Ephrin-A3 mRNA expression upregulated 26-fold in squamous cell lung carcinoma (ii) Expression of ephrin-A3 inhibits EphA2 and EphA3 ligand-dependent activation (iii) EpA5 G518L lung cancer mutation enhances cis-interaction with ephrin-A3	[29, 30]

Ephrin-B2		↑	(i) Expression of ephrin-B2 in A549 lung cancer cell attenuates EphB4 as well as EphA3 ligand-dependent activation	[30]

Ephrin-B3		↓	(i) Silencing of ephrin-B3 in NSCLC line and stabilization of EphA2 via Akt target Ser-897 phosphorylation may promote stability of EphA2 in tumor survival	[23, 28]

**Table 2:** Altered expression of Eph receptors and ephrin ligands in breast cancer.

Eph receptor/ephrin ligand	Preferred molecular interaction	↑/↓ relative to normal tissue	Mechanism	References
EphA2	Ephrin-A1	↑	(i) Ligand-dependent ephrin-A1 activation suppresses migration & ligand-independent activation promotes migration (ii) Herceptin exposure results in Src kinase phosphorylation of EphA2→ activation of PI3K/Alt & MAPK (iii) Ligand-dependent phosphorylation by Erbb2→ amplification of oncogenic Ras/MAPK and RhoA signaling (iv) Anks1 facilitates COPII vesicle loading of ErB2/EphA2 from ER to membrane surface	[12, 13, 34, 35]

EphA4		↑	(i) Higher levels of mRNA EPhA4 → worse prognosis	[36]

EphA7		↑	(i) Higher mRNA of EphA7→ worse prognosis (ii) EphA7–EphA10 heterodimer formation & EphA7–EphA10 nucleus signaling detected in aggressive breast cancer (iii) EphA7–EphA10 cellular signaling detected in normal mammary epithelial cells and EphB6 expressing breast tumors	[36, 37]

EphA10		↑	(i) Higher mRNA of EphA10 → worse prognosis (ii) EphA7–EphA10 heterodimer formation and EphA7–EphA10 nucleus signaling detected in aggressive breast cancer (iii) EphA7–EphA10 cellular signaling detected in normal mammary epithelial cells and EphB6 expressing breast tumors	[37, 38]

EphB2		↑	(i) Localization of EphB2 influence prognostic (1) Worse prognostic in cytoplasmic EphB2 (2) Better prognostic in membranous EphB2 (ii) EphB2/EphB6 heterodimer formation → potential localization of EphB2 based on heterodimer formation	[39, 40]

EphB4	Ephrin-B2	↑ & ↓ (paradoxical)	(i) Ephrin-B2 binding→ activation of Abl-Crk→ downregulation of MMP-2 (ii) Mutant Ephrin-B2 → disrupted E-cadherin expression (1) E-cadherin shedding as a result of ADAM10 (2) EphB regulate ADAM10 activation (iii) EphB4/Ephrin-B2 signaling in MCF7 null PP2A→ activation of oncogenic ERK pathway (iv) ErbB induced expression of EphB4→ inhibition of JAK-STAT & PI3k-AKT pathway	[41–45]

EphB6	Ephrin-B1 Ephrin-B2	↓	(i) EphB6 expression regulated by methylation (1) Inverse relationship between EphB6 and cadherin 17 levels (2) Cadherin 17 activates oncogenic WNT pathway (ii) Kinase null→ phosphorylation via heterodimer formation (iii) EphB6/EphB2 heterodimer (iv) Ephrin-B1 binding→ EphB6/EphB1 (v) Ephrin-B2 binding→ EphB6/EphB4 (1) Heterodimer interacts with c-CBL and phosphorylation of Abl kinase → adhesion promotion (vi) EphB6-EphA2 cross-talking → stabilization of oncogenic ligand-independent EphA2 activation (vii) MDA-MB-231 cells infected with EpB6 revealed decreased transcripts of SMARCC1, eIFC4, eIF4EB2, FKBP1a, FKZBPD5, TRIB1, TRIB3, BMPR1a, and BMPR2	[15, 39, 46–49]

Ephrin-B1	EphB6 Receptor		(i) Binding of ephrin-B1 to EphB6 leads to the formation of heterodimers with EphB1 followed by the phosphorylation of kinase null EphB6 (ii) In vivo in COS-7 revealed Cross-talk of ephrin-B1 activates kinase null EphB6 receptor	[46, 47, 50]

Ephrin-B2	EphB4 Receptor		(i) Interaction of ephrin-B2 results in heterodimer formation between EphB4 and EphB6 with trans phosphorylation of EpB6 and activation of Cbl-Abl pathway leading to proadhesive cell properties in MCF7, MDA-MB-231, and MDA-MB-435 (ii) Protein phosphatase PP involved in ERK pathway activation in ephrin-B2 treated MCF-7 cells (iii) Mutant ephrin-B2 transgenic mice showed aberrant expression of E-cadherin	[42–44, 47, 51, 52]

**Table 3:** Altered expression of Eph receptors and ephrin ligands in gliomas.

Eph/ephrin ligand	Preferred ligand	↑/↓ relative to normal	Mechanism	Reference
EphA2	Ephrin-A1	↑	(i) Overexpression of EphA2 → decreased ERK signaling (ERK crucial in neuronal differentiation in embryonic stem cells) (ii) Ephrin-A1 binding results in ↓EphA2 expression (1) Soluble ephrin-A1 can bind to EphA2 and downregulate EphA2 expression in U251 GMB cells (2) Ephrin-A1 has shown to downregulate focal adhesion kinase in GBM cells resulting in ↓ migration, adhesion, and proliferation (iii) Cross-talking of EphA2 and Akt results in phosphorylation of EphA2 (1) EphA2 expression downregulates Sox2 (iv) Sox2 crucial protein in stem property maintenance	[53–59]

EphA3	Ephrin-A5	↑	(i) Ephrin-A5 binding results in ↑c-Cbl of EGFR receptor → increased degradation of EGFR receptor (ii) GBM cells with overexpression of EphA2, EphA3, and EphB2 killed when exposed to chimeric eA5-PE-C (iii) Expression of EphA3 results in limiting ERK/MAPK activation (1) MAPK signaling drives differentiation of neuronal progenitors	[60–63]

EphA4		↑	(i) Heterodimer complex EphA4-FGFR1 complex → potentiate FGFR mediate downstream signaling to increase proliferation and migration in U251 GBM cells	[64]

EphA5		↑	(i) EphA5 overexpressed in GMB (1) Ligand stimulation with ephrin-A1 did not result in decreased cell proliferation or migration (ii) GBM disease progression correlated with decrease plasma EphA5	[65, 66]

EphA7		↑	(i) Predicator of poor clinical outcome in primary and recurrent GBM patients	[67]

EphA8	Ephrin-A5	↑	(i) Expression of EphA8 → sustained MAPK activity resulting in induced neurite outgrowth in NG108-15 cells (ii) Ephrin-A5 ligand activated EphA8 → no modulation on MAPK activity	[68]

EphB2		↑	(i) Increased expression EpHb2 in human GBM (1) Positive correlation of EphB2 expression and GBM grade (ii) miR-204 posttranscriptional regulator of EphB2 (1) miR-204 reduced in glioma cells due to hypermethylation of host gene TRPM3 (2) miR-204 targets SOX4 and EphB2 (iii) EphB2 has pro-migratory and antiproliferation properties mediated (1) FAK mediates EphB2 migration (2) FAK inhibitors reduced migration in xenograft of EphB2 overexpressing cells (iv) EphB2 activate R-Ras (GTPase)→ decreased extracellular adhesion	[69–72]

EphB4	Ephrin-B2	↑	(i) Higher expression of EphB4 correlated to further progression & worse prognosis of GBM patient	[73]

Ephrin-B2	EphB4	↑	(i) Higher ephrin-B2 associated with worse prognosis (ii) Enhanced migration & invasion with higher expression of ephrin-B2	[73, 74]

Ephrin-B3	EphB2	↑	(i) Possible autocrine or paracrine loop mediated by EphB2 and ephrin-B3 (1) Endogenous ephrin-B3 phosphorylated by EphB2/Fc and exogenous ephrin-B3 phosphorylated (2) Reverse EphB2 signaling dependent on ephrin-B2 in U251 and SnB19 cells (ii) Ephrin-B3 mediates cell migration and invasion through Rac-1 (GTPase) (iii) Rac-1 vital to cytoskeletal organization and plasticity	[75, 76]

**Table 4:** Altered expression of Eph receptors and ephrin ligands in medulloblastoma.

Eph receptor/ephrin ligand	Preferred molecular interaction	↑/↓ relative to normal tissue	Mechanism	Reference
EphA2	Ephrin-A1	↑	(i) EphA2 overexpressed in medulloblastoma samples with vasculogenic mimicry via PI3K (ii) PI3K signaling activates MMP-14. (iii) MMP-14 leads to activation of pro-MMP-2 to activate MMP-2 proteinase. (iv) Active MMP-2 cleave laming 5*γ*2 into promigratory *γ*2′ and *γ*2x fragments which ultimately lead to vascular mimicry formation	[77]

EphB1		↑	(i) Knockdown of EphB1 in DAOY and UW228 human MB cell lines result in ↓ cell proliferation and increased radiosensitization (1) Silenced EphB2 results in decrease cyclin E → decrease phosphorylation of Rb and E21F1 → impaired Chk2 activation → G1 cell cycle arrest (2) Cell cycle arrest results in ↓ levels of proliferating cell nuclear antigen, phosphorylated Akt and total AKT (3) G1 phase is sensitive to radiation (ii) EphB2 and EGFR cross-talk	[78]

EphB2		↑	(i) Overexpression EphB2 in primary medulloblastoma tissue and medulloblastoma cell lines (ii) RNA silenced EphB2 MB cell lines unable to generate invasiveness when stimulated with ephrin-B2 (iii) Knockdown of EphB2 ↑ phosphorylation levels of Erk1/2, MSK1/2, PLC*γ*-1, State1/4/5a, RSK1/2, and cell mobility regulators p27 and paxillin (iv) Ligand-dependent ephrin-B1 enhanced EphB2 expression through downstream activation of p38 and Erk signaling as well as inhibiting mTOR (1) Ephrin-B1 may cross-talk with a serine/threonine phosphatase to dephosphorylate Akt-mTOR (v) Knockdown of EphB2 led to accumulation of medulloblastoma cell arrest in G2/M phase in vitro (1) G2/M phase is most sensitive to radiation therapy	[79, 80]

Ephrin-A5	EphA4 EphA7 EphB1 EphB2	↑	(i) Ephrin-A5 expression associate with medulloblastoma tumor size (1) ND20SmoA1 ephrin-A5 knockout mice had smaller tumor sizes compared to NDS0SmoA1 ephrin-A5 mice (2) Smo constitutive activation suppresses Ephrin-A5 in dorsal midbrain and hindbrain of E12–E15 mice and chicken embryo (3) Ephrin-A5 overexpress in external granule cell layer (medulloblastoma precursor cells) (ii) ND20SmoA1 ephrin-A5 knockout ↓ expression of PCNA and p-Akt (1) PI3K recruits Akt to plasma membrane (2) 3-Phosphoinosito-dependent kinases phosphorylate AKt (iii) PI3K/Akt promotes medulloblastoma growth in a PTEN loss dependent manner	[81–84]

Ephrin-B1	EphB2	↑	(i) Dysregulation of ephrin-B1 promotes oncogenic signaling in medulloblastoma (1) Knockdown of ephrin-B1 in DOAY and D556 cell lines resulted in moderate decrease in phosphorylated EphB receptors compared to knockdown ephrin-B2 indicating possible reverse signaling pathway (ii) Ephrin-B1 knockdown did not reduce phosphorylation of Src suggesting ephrin-B1 may also act outside of the eph/ephrin ax	[85]

Ephrin-B2	EphB2	↑	(i) Knockout of ephrin-B2 in DOAY and D556 cells had lesser decrease of phosphorylated EphB receptors compared to DOAY and D556 Ephrin-B1 knockout lines (ii) DOAY and D556 cells with ephrin-B2 knockout also demonstrated reduction in phosphorylated Src	[85]

**Table 5:** Altered expression of Eph receptors and ephrin ligands in prostate cancer.

Eph receptor/ephrin ligand	Preferred molecular interaction	↑/↓ relative to normal tissue	Mechanism	Reference
EphA1		↓	(i) Lower level of EphA1 transcript levels in primary prostate tumor cells compared to normal prostate epithelium	[86]

EphA2	Ephrin-A1	↑	(i) Highest levels of EphA2 staining in prostatic adenocarcinoma followed by high grade intraepithelial neoplasia with low positive EphA2 staining in benign tissue (ii) EphA2 stimulation with ephrin-A1 resulted in inhibition of PC3 migration	[87, 88]

EphA3		↑	(i) Increase level of EphA3 in androgen independent prostate cancer cells compared to androgen dependent prostate cancer cells (ii) Inactivation of AMP-activated protein kinase (AMPK) resulted in transformation of prostate cancer cell line and upregulation of EphA3 mRNA	[89, 90]

EphA4	Ephrin-A5	↑	(i) cDNA microarray shows EphA4 elevation in prostatic intraepithelial neoplasia and prostate cancer (1) SiRNA knockout of EphA4 resulted in decrease cell viability and colony formation (ii) EphA4 stimulation with ephrin-A5 resulted in inhibition of PC3 migration (iii) EphA4 may be regulated by ERBB3/HER3 (1) Knockdown of ERBB3 in DU-145 resulted in downregulation of EphA4	[88, 91, 92]

EphB2		Mutant expression of inactive EphB2 in prostate cancer	(i) Two mutations in extracellular part of EphB2 and six mutations in the intracellular part of EphB2 (1) Extracellular mutation: R199H, A279S (2) Intracellular mutation: D679N, 2139+2T→C, Q723X, T909M, K1019X, and 3051delA (ii) Loss of EphB2 in primary prostate tumor cells compared to normal prostate epithelium cells	[86, 93]

Ephrin-A1		↓	(i) Higher Gleason score correlate with lower levels of ephrin-A1	[94]

Ephrin-A5	EphA4 EphA7 EphB1 EphB2	↓	(i) Proteomic analysis of LnCaP cell culture media demonstrated increased levels of ephrin-A5 post androgen exposure (ii) Lower levels of ephin-A5 correlated with shorter predicated survival time	[95, 96]

**Table 6:** Altered expression of Eph receptors and ephrin ligands in colorectal carcinoma.

Eph receptor/ephrin ligand	Preferred molecular interaction	↑/↓ relative to normal tissue	Mechanism	Reference
EphA1		↓/↑	(i) Heterogeneous expression with both elevated/depressed expression depending on stage of CRC (ii) Low EphA1 expression strongly correlates with poor survival (*P* = 0.02) (1) EphA1 overexpression more prevalent in stage II CRC compared to stage III CRC (*P* = 0.02) (iii) EphA1 knockdown promotes spreading and adhesion of HRT18 cells (1) Activation of ERK and c-Jun NH2-terminal kinase (JNK) signaling pathway with EphA1 knockdown (iv) EphA1 down regulation via epigenetic silencing (1) CpG islands in promoter region of EphA1 gene (2) EphA1 reexpression after demethylating agent treatment	[97–100]

EphA2		↓/↑	(i) Heterogeneous expression with both elevated/depressed expression depending on stage of CRC (1) Up to 5-fold increase of expression in CRC versus normal tissue (ii) Higher EphA2 expression correlate with poor survival in stage II/III colorectal cancer tissues (1) Downregulation of EphA2 via RNAi or recombinant EFNA1 suppressed migration and invasion in KRASMT CRC cells (iii) EphA2 regulated by KRAS-driven MAPK and RalGGS-RalA pathway	[97, 101]

EphA3		↓	(i) Decreased in CRC tumor specimens (ii) Overexpress, decreased expression and ectopic Eph3 expression had no effect on tumorigenesis in xenografts or mouse models	[97]

EphA7		↓	(i) EphA7 down regulation via epigenetic silencing (1) CpG islands in promoter region of EphA7 gene	[97, 102, 103]

EphB2		↓	(i) Reduced in CRC compared to normal colorectal tissue (1) Higher EphB2 correlates with better prognosis (ii) Expression regulated by transcription p300 via TCF/B-catenin pathway	[104, 105]

EphB3	Ephrin-B1	↓	(i) Reduced EphB3 in advanced Duke's stage tumor specimen (ii) EphB4 overexpression resulted in inhibition of HT29 growth and inhibit epithelial to mesenchymal transition (1) Activation of zonlua occludens-1, E-cadherin, and plakoglobin	[106, 107]

EphB4		↑	(i) Stable EphB4 overexpression in SW480 resulted in increased growth and invasion (ii) Expression regulated by transcription CBP complex via TCF/B-catenin pathway	[97, 104, 105, 108, 109]

EphB6		↓	(i) Lower EphB6 correlates with poor cell differentiation, advance disease, metastatic spread, and poor prognosis (ii) Xenograft and mouse models demonstrate sole loss of EphB6 to be insufficient to trigger tumorigenesis	[110, 111]
